# Conceptualizing and Measuring Violence: A Feminist Critical Measurement Analysis of Gender-Based Violence Research in a Government Policy-Based Setting

**DOI:** 10.1177/10778012251320586

**Published:** 2025-03-12

**Authors:** Lucy Thompson, Tanya Frances, Emma Turley, Lisa Lazard, Lois Donnelly

**Affiliations:** 1 3078 Michigan State University, East Lansing, MI, USA; 2 5488 The Open University, Milton Keynes, UK; 3 6939 Central Queensland University, Brisbane, QLD, Australia; 4 8709 University of Worcester, Worcester, UK

**Keywords:** gender-based violence, intersectionality, feminist quantitative research, policy, qualitative research methods

## Abstract

Feminist methodological advances in gender-based violence research have yet to be comprehensively integrated into policy-based research. This article discusses methodological concerns identified when reviewing the UK government's public consultation for its 2021 Tackling Violence Against Women and Girls Strategy. We apply intersectional feminist perspectives and critical measurement analysis, and discuss two areas of methodological concern: issues with conceptualizations of violence, and issues with the measurement of public experiences, attitudes, and opinions, and implications for the accuracy of the survey. In response, we consider feminist research practices that could address these issues and support feminist goals in policy-based gendered violence research.

## Introduction

Following the success of early women's and feminist activism in placing the prevention of gender-based violence on national and global policy agendas (Basu, 2014), there has been growing recognition of the need for “methodologically rigorous research…to guide the formulation and implementation of effective interventions, policies, and prevention strategies” (Ellsberg & Heise, 2002, p. 5). To this end, organizations such as the World Health Organization have published research methodology guides for those working in this area to develop methodologically sound and comparable findings related to gender-based violence. Feminist scholarship has been particularly instructive for developing nuanced considerations of research practices and processes that work toward the above goal by centering survivors in the development of knowledge. This work has also shown how methodological design, implementation, and data interpretation can perpetuate problematic gendered power inequalities (Leung et al., 2019; Nyklová et al., 2023).

Typically, these considerations have been examined and discussed in relation to academic research. This article explores these considerations in relation to governmental and policy-driven research. Specifically, we focus on the use of research by the UK government to inform their most recent Tackling Violence Against Women and Girls (VAWG) Strategy (HM Government, 2021). We (the authors) were invited by the British Psychological Society (BPS) to review and respond to the UK government's call for evidence as academic consultants with specialisms in this area of research and practice. All authors are members of the Intersectional Violences Research Group, an international collective of academic feminist psychologists with related research interests. We take a critical feminist psychological approach to the study of a range of violences including domestic and family, sexual, and epistemic violence. In this work, we apply feminist intersectional perspectives to understand dynamics and functions of power, examine the constructions of difference that legitimize this power, and promote social change and policy reform. In the current analysis, we apply this perspective to identify and consider some methodological concerns that arose from our engagement with this particular national consultation project.

The UK government releases strategy documents on various issues which aim to set out the government's approach to addressing these issues in the UK, including the actions they will take to ensure progress in this area. These strategies are often informed by consultations with the UK public, who may be seen as “experience experts,” and professionals who work in the area under investigation, either through practice or research. These consultations are often presented as online surveys. The consultation was framed as a “call for evidence” and used a survey tool to “to hear directly from the public on tackling violence against women and girls” (HM Government, 2021, p. 3). The ultimate goal of the research was to generate “evidence” to inform government strategy. This public call is perhaps not surprising in light of more visible public support for survivors of violence, particularly since the rise of #MeToo activism (Maier, 2023). The research also invited responses and submissions from academics and others with expertise and interest in the area of VAWG. Therefore, this research can also be seen to reflect aspirational moves within political cultures to embed policy making in an evidence base (Cairney, 2016).

While strategy is distinct from policy, the two are enmeshed. UK Parliament presents clear distinctions and definitions on this basis: “Strategy is not policy, but is the means of effecting it” (UK Parliament, 2010, para. 4). However, this distinction should not be mistaken for rigid bifurcation, as there is also clear recognition of a “symbiotic relationship between the two” (UK Parliament, 2010, para. 4). Indeed, with policy defined as the “end,” and strategy defined as the “ways and means” (UK Parliament, 2010), the two are viewed as inextricable. Strategy therefore constitutes an important mode of action in the form of policy implementation, and UK Parliament notes that policies are ineffective without strategy. This call for evidence therefore suggests aspirations to embed policy *implementation* in an evidence base as well. While this goal is important, a body of work has pointed out that the use of “evidence” in practice is a highly complex endeavor that is complicated by, for example, different political pressures, agendas, and incentives for engagement between researchers and policy makers (Malbon et al., 2018; Newman et al., 2016). Further critical attention to the “evidence” informing government strategy, and how this is generated, is therefore warranted.

In this article, we will begin by outlining the feminist approaches and methodological concerns that ground our analysis. Specifically, we will discuss feminist intersectional perspectives (Cooper, 2015) and critical measurement analysis (McClelland et al., 2020). Based on this, we will then turn to the methodological considerations we identified through our engagement with the government's consultation, and specifically their research design and approach, including the conceptualization(s) of violence embedded in their data collection tools, and methodological issues relating to the measurement of attitudes and understandings of violence. In response, we critically consider implications for quantitative policy-based research in this area drawing on feminist perspectives, and outline some feminist methodological contributions that could support research methods and practices in policy-related gender-based violence research. Through this analysis, our goal is to highlight the methodological issues and implications that can arise in the process of generating “evidence” to inform gender-based violence policy and its implementation.

### Feminist Approaches to Gender-Based Violence Research

Doing gender-based violence research is fraught with methodological, conceptual, and ethical complexities that have been discussed and navigated by scholars working with different disciplinary, theoretical, and methodological frameworks (Beetham et al., 2021; Sprague, 2016). Specifically, a vast domain of feminist research and activism has consistently drawn on broader feminist concerns around these complexities as they relate to gender-based violence, driving efforts to understand violence within systemic patterns of inequality, and maintaining the vital role of feminism in public advocacy efforts.

Whilst feminist research can vary widely in its methodological approach and focus, it remains informed by a broader shared approach and epistemology. For instance, feminist research (regardless of method) aims to be reflexive, focused on gender, and committed to the political aim of social change (Scott, 2010). Feminist researchers also accept that the researcher is inescapably located within their own research (Fleischman, 1998; Letherby, 2003) and acknowledge that there are power dynamics within the research process itself (Fonow & Cook, 2005; Letherby, 2003). Feminist research, diverse as it is, brings these considerations together through a shared commitment to questioning and transforming gender inequities. This commitment is embedded in established feminist critiques of methodological approaches that have broadly obscured, minimized, or trivialized women's experiences; particularly in relation to gender-based violence and the power relations and inequities that shape this (e.g., Damant et al., 2008; Eriksson, 2013; Gill, 2018; Lazard, 2020). In these critiques, there is an important recognition of the epistemic exclusions that have been brought to bear in knowledge production practices that are hostile toward feminist work. Indeed, as DeKeseredy (2011) notes in relation to psychological research, feminist scholarship has suffered at times vitriolic and inaccurate criticism by mainstream psychological researchers. Such misguided criticism inflates a false impression that feminist and mainstream research is starkly divided, and that feminist researchers are somehow less methodologically rigorous than their mainstream counterparts. DeKeseredy (2011) argues that, contrary to such criticism, many advances in methodological approaches to gender-based violence are attributable to feminist scholarship. Offering the example of methodological developments in large-scale victimization survey research, DeKeseredy shows how the use of feminist and “women-centered” approaches yields higher rates of prevalence than conventional surveys (p. 300).

More recently, feminist conceptual and methodological insights have contributed to an explicit recognition that gender-based violence “cannot be understood properly or eradicated without a feminist analysis to underpin research, policymaking, and programming” (Leung et al., 2019, p. 430). We agree with this position, and argue further that this needs to be grounded in a specific recognition of the capacity of *intersectional* feminist thought to ask important questions and guide understandings of gender-based violence in these contexts. This is because intersectional feminist thought enables critical analysis of who is rendered legible, and illegible, in particular contexts (Cooper, 2015). This type of analysis is especially important in relation to gender-based violence because it is intersectionally patterned, and this has not been consistently recognized in research, policy, and activism (Crenshaw, 1991, 2023).

In policy-related contexts, gender-based violence researchers have examined “gendered intersectionalities” (Hearn et al., 2016, p. 551) to show how a range of inequalities are made visible and invisible in state policy on violence. On this basis, the authors advocate for the recognition of multiple inequalities in policy. In line with Crenshaw's original conceptualization of intersectionality (Crenshaw, 1989), Cooper (2015) discusses intersectionality as an analytical framework rooted in legal and policy analysis. In doing so, Cooper (2015) draws attention to “unfortunate” and “egregious” (p. 389) interpretations and applications of intersectionality as a feminist theory of identity, contrasting this against the original conceptualization, which “allowed the recognition of the black female subject within judicial structures of power, where she had heretofore remained invisible and illegible, and thus unable to obtain any kind of justice” (p. 390). Here, there is an explicit focus on structural identities: “The law conceptualizes people through the structural identities of gender, race, sexual orientation, or national origin” (Cooper, 2015, p. 390). On this basis, Cooper (2015) argues that the focus of intersectional analysis should be on developing and sustaining critiques of the power arrangements that render particular identities invisible and therefore illegible. In line with this analysis, we were concerned with examining who and what was made legible and illegible in this public survey, and the implications of this for subsequent policy development and implementation. We will now situate our work in relation to key methodological concerns that have been raised by those working in the area of gender-based violence, before discussing our analysis of the consultation survey.

### Methodological Concerns in Gender-Based Violence and Policy-Related Research

As Ramazanoglu and Holland (2002) argue, epistemology, ontology, ethics—and the production of knowledge in relation to the consequences it has for accountability and power—need to be properly considered in methodological decision-making, planning, and practice. For this reason, feminist researchers argue that it is important to consider how methods are used and for what purpose (Ramazanoglu & Holland, 2002). Existing literature draws attention to a range of interwoven methodological concerns that arise for those engaged in gender-based violence research. These concerns have largely been framed around the recognition of situational power relations and imbalances in research processes. A wealth of feminist research has discussed, critiqued, and attempted to address these power imbalances through their methodologies; for instance, by seeking to construct knowledge through reflexive and relational processes (Hesse-Biber & Piatelli, 2012; Leung et al., 2019). In this work, creating space to allow participants to tell nuanced stories about their experiences has been a key priority (Hesse-Biber & Piatelli, 2012).

Proponents of qualitative, critical, and feminist research (that seeks to center these nuanced stories) have critiqued quantitative research in psychology on the basis that it does not allow space for such nuance (Creswell & Poth, 2016). These critiques have also raised important concerns about claims of objectivity and standardization in quantitative work, engendering doubts about whether these methods can produce socially valid and progressive knowledge (Tafreshi et al., 2016). However, such arguments tend in fact to be criticisms of positivist epistemic practices, and should not necessarily be confused with methods themselves (Sprague, 2016; Stauffer & O’Brien, 2018). Indeed, as Sprague (2016) notes, epistemology and method should not be conflated. Quantitative methodologies can be (and are) used within feminist frameworks (Hesse-Biber & Piatelli, 2012), with an emphasis on producing socially valid and progressive knowledge while avoiding the reproduction of patriarchal ways of knowing (Reinharz, 1992). Leung et al. (2019) suggest that feminist quantitative research is possible if a feminist lens and corresponding principles are used throughout the research process, from design to publication. These principles include being ethical, collaborative, transformative, intersectional, accountable, and accessible (Leung et al., 2019). Thus, quantitative research methods are not necessarily at odds with feminist goals. This is an important observation, because large-scale policy research often employs quantitative methods. However, such research is not typically designed and implemented in line with feminist goals.

The UK government consultation discussed in this paper employed a survey design with largely quantitative items. There are several strengths to this method from the perspective of policy makers. First, as one of the most popular and widely employed quantitative methods, surveys can be an extremely effective tool for social change because broad patterns of experience generated by large samples and easy-to-read numerical information can effectively influence policy (Miner et al., 2014). In addition, whether accurate or not, quantitative survey research may be viewed as the most “objective” paradigm by policy-makers, and is thus more likely to be valued as legitimate when building “evidence-based” policies (Malbon et al., 2018). This may also explain why policy makers themselves use survey research in their own investigations, as with the UK government consultation discussed in this paper. Put simply: surveys travel further, both literally and ideologically. However, again, important feminist considerations are often missing from survey design and implementation in this context. This is despite long-standing efforts to promote survey research based on feminist principles. For instance, Reinharz (1992) advocates for survey design that goes beyond traditional methods and argues that surveys should not only aim to generate data but also to challenge existing power structures, question and challenge dominant narratives, and center the voices of marginalized groups, including women. She highlights the importance of writing survey questions that are sensitive to gender and power dynamics and inclusive of diverse experiences. This involves avoiding biased language, offering response options that reflect a range of identities and experiences, and framing questions in a way that acknowledges the social context in which respondents live (Reinharz, 1992). In addition, Reinharz (1992) highlights how traditional survey sampling strategies may exclude certain groups of people—particularly those who are marginalized and minoritized—and calls for targeted, purposive sampling techniques that prioritize diversity. It is also argued that quantitative analyses, including survey item analysis, can be conducted through a feminist lens that incorporates explorations of power dynamics, inequalities, and the intersections of gender with other social categories such as race and ethnicity, social class, and sexuality (Reinharz, 1992).

More recently, specific feminist attention has been paid to the inherent meanings and assumptions constructed in surveys and survey items. Critical measurement analysis (McClelland et al., 2020) draws on feminist psychological principles to “(1) identify patterns in survey items used across a wide range of studies, (2) identify potential biases in existing items, and (3) identify areas that have been consistently overlooked in survey research” (McClelland et al., 2020, p. 9). This analytical method aims to identify how research topics are constructed and rendered legible by researchers, what assumptions underpin the items developed by researchers, who is rendered legible (and illegible) by these items, and how they are represented by these items. Critical measurement analysis is important because researcher assumptions play a central role in the questions they ask. For instance, in their analysis of survey item banks used in public abortion attitude surveys, McClelland et al. (2020) identified a “*dispersion of bias*” (p. 19), through consistent repetition of historical stereotypes linking “poor” mothering and Black women. On this basis, McClelland et al. identify three main concerns associated with the assessment of abortion attitudes, which can be applied more broadly. First, they argue that these biases and stereotypes constitute a form of inaccurate measurement. Second, they argue that such negative biases and stereotypes render the subject(s) of the research illegible; or unable to be fully or accurately known (and known about). Third, they argue that research and survey items themselves actively contribute to the reproduction and proliferation of these negative biases and stereotypes through public circulation, media reporting, and other outlets. In response, McClelland et al. (2020) urge researchers to pay critical attention to the biases, cues, and stereotypes surrounding particular topics when designing and administering surveys: “Without prioritizing investigations of the tools that are used—in other words, how people interpret, respond, and imagine worlds in response to the questions that are asked—researchers risk repeating racist and sexist tropes and calling it psychometrically sound” (p. 20). As well as providing a critical analytical approach for survey design, this work also aligns with intersectional feminist theory in its focus on who and what is rendered legible, and how. Thus, critical measurement analysis lends itself closely to feminist intersectional analysis of policy-related research tools, especially because it allows for an understanding of who and what comes under the view of policy, how they come under this view, and who does not.

It is typically assumed that advances in methodological approaches of the type discussed here are integrated into policy-related research by academics and others with research expertise and critical awareness of these kinds of methodological concerns. However, based on our experience, we argue that this is not necessarily the case. To support this argument, our review of this call for evidence engages critical measurement analysis to examine the assumptions underpinning survey items, and consider the implications for policy and policy-related research in the context of gender-based violence.

## Reviewing the UK Government's VAWG Public Consultation Survey

We conducted this review of the UK government's public consultation survey leading up to the publication of their 2021 Tackling VAWG Strategy (HM Government, 2021) as part of our existing work with the Intersectional Violences Research Group. The public survey and introductory material provided on the government's website stated that the aim of the survey was to collect “[the] public's views about violence and crimes that predominantly affect women and girls.” We were asked to provide a response to the consultation on behalf of the BPS. To complete this review, we were provided with the government's initial call for evidence and the public consultation survey. Upon our initial reading of these materials, we were troubled by the content of the public consultation survey, its underpinning assumptions, and the implications of these. As such, we conducted a more detailed review of the survey to better develop our understanding of what troubled us about its content. To guide our review, we applied the principles of critical measurement analysis (McClelland et al., 2020).

To begin, each author individually reviewed the survey. We then met as a group to discuss initial findings. Through this initial review, it became clear that there were numerous issues with the survey's conceptualization of violence, and the items developed to measure public attitudes about this. In response, we returned to the survey to complete a comprehensive analysis of the content. Here, we carried out a critical measurement analysis (McClelland et al., 2020), which applies the principles of thematic analysis (e.g., Terry et al., 2017) to generate qualitative insights into the meanings apparent in the items. To accomplish this, we read the survey in depth several times and made detailed notes on our initial observations, before identifying specific meanings inherent in the survey. For example, we identified specific *types of violence* that were provided as examples (such as honor-based violence, stalking, female genital mutilation, and specific forms of sexual harassment, such as upskirting). Next, we reviewed these specific meanings (or codes) and grouped similar meanings together to generate preliminary themes. For example, we grouped all of the different *types of violence* we had identified together. We then refined these themes by examining the “central organising concept,” or core meaning, tying each group together. For example, in relation to “types of violence,” we noted that the examples were all very extreme. This led us to generate the theme: “Sensationalised conceptualizations of violence.” We repeated this process for each of these groupings until we had a series of internally and externally distinct themes. In the following sections, we discuss six key themes we generated through the analysis, with examples from the survey to illustrate these themes. We begin by discussing four overarching themes pertaining to the conceptualizations of violence that were inherent in the survey. We then turn our attention to two further themes that raised concerns over the measurement of violence and the items themselves. Throughout, we also consider the intersectional implications of the meanings inherent in the survey, and consider who and what is rendered legible (and illegible) in this public consultation.

## Analysis of the Public Consultation Survey

Our analysis generated six themes which fell under two broad categories. The first category pertains to conceptualizations of violence constructed in the survey and its items. The four themes situated under this category construct violence in line with specific and distinct assumptions about what violence is, how it is done, and who experiences it. The first theme refers to recurring meanings that constructed violence as a depoliticized *act-based* phenomenon. The second theme refers to recurring meanings that imagined violence within a *carceral* framework while ignoring the limitations and harms of carceral systems. The third theme refers to recurring meanings that conceptualized violence in a *sensationalized* manner while ignoring less “extreme” forms of violence. Finally, the fourth theme refers to recurring meanings that constructed violence in line with dominant assumptions about *cisheteronormative* gender and relationship paradigms.

The second broad category pertains to the validity and accuracy of the items in the survey, and their implications for the measurement of violence. Specifically, the two themes situated under this category identify two distinct and specific forms of bias underpinning the survey items. The first theme refers to recurring meanings that *reproduced myths about violence* in the survey items. Conversely, the second theme refers to recurring meanings that *contested the realities of violence*. We will now discuss these two broad categories, and the themes therein, before considering the implications for research and policy.

## Conceptualizations of Violence

### Act-Based Conceptualizations of Violence

In our analysis, we identified problematic conceptualizations of violence which resonated with existing feminist psychological concerns over the way(s) that gender-based violence is interpreted and understood (e.g., Lazard, 2020). We identified several instances where violence (in particular, domestic abuse) was framed as incident or act-based, rather than a pattern of repeated behavior aimed at controlling a partner (Stark, 2007). For instance, survey participants were asked to determine whether they thought certain acts or types of violence were crimes. Examples included:

A partner or family member hitting their partner or someone else in the family

Someone sending abusive texts and messages online

A person having sex without agreeing to it

Strangling someone as a way of controlling their behaviour.

The incident and act-based conceptualization of violence, including the above examples of domestic violence, sexual violence, harassment, and online abuse, is narrow and relies on individualized assumptions that do not account for the structural, relational, or intersectional dynamics that underpin and enable violence (Burman & Brooks-Hay, 2018; Segalo & Fine, 2020; Stark & Hester, 2019). To draw on domestic abuse as an example, it is well recognized that domestic violence is characterized by controlling power dynamics which cannot be captured by counts of incidents (Walby & Towers, 2017). The promotion of an incident and act-based model appears to overlook a wealth of existing research that highlights this point, and risks perpetuating inaccurate and potentially harmful misconceptions and myths around domestic and sexual violence (e.g., that it is act-based). This act-based conceptualization also overlooks conceptualizations of gender-based violence as “a form of power, inequality and control” (Hearn et al., 2016, p. 552), rendering these power dynamics, their disparate impacts, and those who are impacted along these lines, illegible. For example, in her consideration of structural intersectionality, Crenshaw (1991) outlines the burdens of class and gender oppression in the lives of women of color, such as poverty and childcare responsibilities, which are compounded by racially discriminatory practices. These conditions mean that women of color who experience violence face unique obstacles. An act-based conceptualization of violence renders these conditions, and the experiences of women of color within them, illegible. This has implications for public understandings of violence, and concerningly, implications for members of the public who have experienced violence. If the conditions that give rise to differential experiences of violence are not accounted for, and those experiences are rendered illegible, those who experience violence are also rendered illegible in research, policy, and practice. Therefore, when conceptualizing violence, it is important for researchers to ensure that violence is represented in a range of ways that individuals can identify and identify *with*. Failure to do so can limit the scope of *how* people can report experiences of violence, and subsequently how policy can address this. Therefore, it is imperative that policy makers and researchers engage with the wealth of existing scholarship relating to more nuanced and complex conceptualizations of gender-based violence (e.g., Gavey, 2018; Lazard, 2020; McGlynn & Westmarland, 2018; Nicolson, 2019; Segalo & Fine, 2020) and consider the broader implications of perpetuating problematic messaging around violence. Overall, a focus on the broader power relations shaping experiences of violence would expand act-based conceptualizations of violence and allow researchers to develop intersectional conceptualizations of violence to guide research and policy.

### Carceral Conceptualizations of Violence

Notions of criminalization were central to conceptualizations of violence underpinning the survey. As such, the survey was framed by a dominant carceral approach to gender-based violence. The act-based model of violence used in the survey invited participants to identify which “acts” were—and which were not—crimes. The first question in the survey asked members of the public: “Do you think these are crimes? Tick if you think they are” and listed several forms of violence (e.g., “a person filming up someone's skirt without permission” and “someone sending abusive texts and messages online”). This question asks participants to indicate if they know whether the listed examples of violence are crimes. Members of the public cannot meaningfully answer this question without specialized legal knowledge about which examples do or do not constitute existing criminal offences. If the intention was to ask participants whether they think these examples “should” be crimes, the wording would need to reflect this. As it stands, these items are designed to measure the participants’ knowledge about crimes, which could vary drastically according to a range of factors that are not captured by the survey. This is further complicated by the fact that, after this question, participants are asked an open-ended question: “Are there any other behaviors you think should be a crime?” Here, inconsistencies between the two parts of the question (i.e., what “are” crimes vs what else “should be” a crime) call into question the validity of these items.

There were also inconsistencies in the framing of other questions, which revealed an underpinning assumption that these behaviors were indeed crimes. For example, in other parts of the survey, the same behaviors were presumed to be crimes. For instance, when asking members of the public if they had personally experienced VAWG, the question was: “Have you been a victim of any of the crimes mentioned in this survey?”. Additionally, another question asked participants to “Tick the 3 most important things the government must do to stop violence against women and girls” where almost all of the options mentioned criminality or crime in some way, such as:

Make sure the public know about these crimes

More programmes for criminals to change their behaviour and stop them committing crimes again

Longer sentencing for criminals

Reduce the time that victims have to wait to make court cases happen.

All of these items refer to actions within criminal justice systems, whether this is through education about crime, programming, sentencing, or court processes. This narrow definition privileges one specific (and often limited) way of responding to violence, obscuring the everyday realities of violence and the possibilities for responding. Dominant carceral conceptualizations of violence therefore carry conceptual limitations, which make other understandings of violence and forms of action unavailable. For example, the survey narrowly prioritized criminalized violence rather than an array of different (criminalized and non-criminalized) forms of violence. The focus on criminalization also failed to recognize limitations with carceral systems, such as the fact that reporting and conviction rates for sexual assault, domestic violence, and rape in the UK are already very low (HM Government, 2021), and the fact that criminal justice responses can be limited and are not always the safest or most appropriate solution. For instance, evidence shows that for many survivors of violence, the criminal justice system re-traumatizes and re-victimizes complainants, and the system itself can be violent, traumatizing, and oppressive (MacQueen & Norris, 2016). Such constructions of violence are problematic for survivors because they fail to engage with or capture the everyday dynamics and realities of gender-based violence and systemic failures to address it. An uncritical carceral approach also fails to account for active practices of criminalization, where racism, classism, and other forms of oppression have created racial, ethnic, and class-based disparities in rates of incarceration. This approach privileges a carceral framework that has historically failed those who experience violence, while doing violence to those deemed “criminal” and affording others the privilege of doing violence without reprise. As noted by McClelland et al. (2020), such narrow framing presents a one-sided consideration of the topic at hand. Given the issues we have discussed in relation to carceral responses to violence, there should be opportunities to consider alternative ways of conceptualizing the “problem” of violence, which could include, for example, alternative modes of justice and change such as restorative justice, community-based justice, and specific forms of structural change. Without a range of alternative options, participant non-response could be taken as an indication that no intervention, criminal sanction or otherwise, is needed. However, such a conclusion cannot be drawn when alternatives are not included in the question format.

There may also be serious implications for those who have experienced violence who participate in surveys such as this. Social “problems” are constructed through their representation in public spaces, including via institutional documents. When considering research from the perspective of participants, a key consideration for this survey, and for research more broadly, would be to acknowledge issues of failure in criminal justice practices rather than reinforcing carceral frameworks uncritically. A focus on individual perpetrators without the same focus on the problems with criminal justice practices only serves to individualize the issue of violence and fails to address relational and systemic issues with criminal justice approaches. Moreover, an expansion of criminalization without input from those who have experienced violence on what constitutes “justice” reproduces the very systems and practices that have failed them. This includes understanding how differential experiences and understandings of “justice,” and those who deserve and receive it, are intersectionality shaped and produced. As Cooper (2015, p. 390), reminds us, intersectional analysis “allowed the recognition of the black female subject within judicial structures of power, where she had heretofore remained invisible and illegible, and thus unable to obtain any kind of justice.” Therefore, intersectional understandings of disparities in the allocation of justice should be central to work or research that considers violence within carceral systems.

### Sensationalized Conceptualizations of Violence

In the survey, different types of violence were generally conflated using the term “violence against women and girls.” However, where specific examples were provided in the survey, they were narrowly conceptualized and frequently sensationalized (e.g., violent sex, physical violence, female genital mutilation, honor-based violence, and strangulation). Indeed, the survey was framed by the following examples of violence: “This can include sexual offences, domestic abuse, stalking, upskirting, female genital mutilation, ‘honour-based’ abuse as well as many others.” Overall, this sensationalized conceptualization of violence fails to engage with the realities of gender-based violence in which the most common incidents tend to be less “extreme.” Expert scholarship has also consistently drawn attention to the complexities of different types of violence. For example, the processes and practices underpinning coercive control are substantially different to incidents of strangulation, or upskirting on the street. Therefore, the logic of conflating distinct forms of violence is highly questionable, as is the practice of cherry picking only the most sensationalized examples. In terms of measurement alone, the conflation of these categories along with the use of only extreme examples in the design of this survey would likely lend itself to response bias where participants may orient or anchor their responses to these more extreme forms of violence rather than thinking about a continuum of violence. This means that many forms of common, yet less extreme, violence may not be as adequately considered and captured by the survey, and therefore may not be addressed in the government's VAWG strategy.

In addition, these sensationalized examples specifically function to render certain racialized forms of violence (“honour-based” violence and female genital mutilation) legible, presumably making those who do this violence legible as well. Coupled with the carceral approach, this focuses on and prioritizes the criminalization of very specific forms of violence, as opposed to more common, yet less extreme, forms of violence. This marks out and criminalizes particular groups, in this case reflecting anti-Muslim and Islamophobic discourses which operate to racialize crime, and especially sexual violence, in the UK (Cockbain & Tufail, 2020). Applying an intersectional feminist analytic frame here can reveal a politics of *legibility*, where legibility can be used to mark out and problematize particular (already-stigmatized) groups, raising intersectional questions over *who* is constructed and depicted as violent. While it is important to address these forms of violence, they are not the only specific forms of violence that occur. However, they are singled out in such a way that reinforces racist rhetoric and attention. Again, then, in line with McClelland et al. (2020) the survey further contributes to the circulation of negative biases and stereotypes, when efforts should be made to avoid this. Therefore, it is imperative that researchers and policy makers make efforts to identify and avoid the reproduction of biased representations of violence, particularly when this contributes to racist rhetoric, surveillance, and stigmatization.

### Cisheteronormative Conceptualizations of Violence

In the survey, violence was conceptualized within a cisheteronormative framework, and the experiences of anybody but heterosexual and/or cisgender individuals or groups were not specifically reflected in the examples of violence presented. This conceptualization was mainly evident in the imagery used in the survey: The main depiction of violence showed a white man raising his fist to a white woman. She was then shown again as a victim of violence later in the survey. Given that it is estimated that at least 3.7% of the population of England and Wales identify along the LGBTQ+ spectrum (Office for National Statistics, 2023a, 2023b), excluding or failing to specifically ask about the personal experiences of these groups will result in a less nuanced, inclusive, and appropriate strategy. This exclusion becomes even more pronounced considering that members of LGBTQ+ communities are more and disproportionately at risk of violence, including domestic and sexual violence, in comparison with their cisgender and heterosexual peers, with trans and bisexual women being the worst affected (Bolam & Bates, 2016; Messinger & Koon-Magnin, 2019; Semprevivo, 2021). These risks are further exacerbated at the intersections of race and ethnicity, where LGBTQ+ people of color, particularly Black and Latine trans women, face higher rates of violence (Westbrook, 2023). Research illustrates that Black transgender women are disproportionately affected by both interpersonal violence and systemic discrimination, often resulting in compounded vulnerabilities (Messinger et al., 2022; Sherman et al., 2022). In addition, LGBTQ+ people of color regularly encounter additional barriers to accessing support services, which are often structured around the experiences of white, cisgender individuals, leaving racially minoritized LGBTQ+ survivors of violence further marginalized (Guadalupe-Diaz & Jasinski, 2017; Sherman et al., 2022).

By excluding LGBTQ+ people's experiences of violence and centering cisgender and heterosexual experiences as normative, the formulaic story of domestic and sexual violence is perpetuated where a cisgender man is the abuser and a cisgender woman is the victim within the relational context of a heterosexual relationship (Kranick, 2015). Drawing on these stereotypes not only perpetuates myths about which groups experience and perpetrate violence, but also closes down discussion about other types of violence and forms of victimization and positions those who are outside of this formulaic narrative as illegible and as Other. As Teo (2010) argues, epistemological violence occurs through the interpretation of the Other as problematic, which has negative impacts on the Other through misrepresentation or exclusion. Most violence response strategies are already designed for white, middle class, heterosexual, cisgender women, and do not recognize or meet the needs of marginalized people (Gill, 2018). By excluding particular marginalized groups in this way, the strategy was unlikely to differ. As a consequence, the public survey privileged the experiences of certain groups while erasing others, which is an act of epistemic power exercised by the government through their survey design. However, this becomes epistemic violence through the exclusion of the Other in the survey and therefore the broader strategy, which impacts support and services for those against whom violence is enacted. This is an example of illegibility in action, where those who are rendered illegible are prevented from obtaining justice (Cooper, 2015). The politics of voice become an intersectional issue here. This refers to “who gets to speak, but also who gets listened to, authorised, publicised and legitimated” (Fraser & Taylor, 2022, p. 226). These arguments are not new (Cook et al., 2022). Feminist scholars have argued for decades that power relations operate in various contexts including in research practices, meaning that some groups are advantaged, while others become silenced (Burman, 2016; Houston & Kramarae, 1991; Skeggs, 1999). Our review of the UK government's public consultation survey shows that these arguments are still needed, specifically in the context of large-scale public consultation research practices. It is therefore imperative that researchers in policy-based contexts resist the reproduction of epistemic exclusions, injustices, and violences that render those who are marginalized illegible.

## Measuring Violence: Item Validity and Accuracy

Upon the release of the survey, the government's website stated that the aim of the research was to inform how the government would produce a new strategy to ‘tackle' VAWG, and that survey responses would be used for this purpose. In addition to the issues we have discussed in relation to the conceptualizations of violence in the survey, we identified issues relating to particular survey items that raised questions over the validity and accuracy of these items. This raised questions over the capacity of the survey to meet its aims. In this section, we discuss two themes that raise questions about the measurement of violence, the accuracy and validity of the survey items, and the “evidence” produced.

### Reproduction of Myths about Violence

We found that rather than asking objective questions to inform policy, several items instead encouraged participants to evaluate myths about violence.

This included statements such as:

Domestic abuse always involves physical violence

A lot of people who say they have been raped are making it up

People may be partly responsible if they get raped when they have had too much to drink.

These items repeat myths surrounding violence, and invite responses that rely on inaccuracies and biases. Specifically, two of these items (“A lot of people who say they have been raped are making it up”; “People may be partly responsible if they get raped when they have had too much to drink”) reinforce rape myths. Myth-based items are usually used to measure victim-blaming attitudes rather than knowledge and understandings of violence. If it is the case that these items are intended to measure victim-blaming attitudes, it is unclear how this information informs the government strategy. For instance, even in the final strategy, myths are only mentioned in relation to victims stating that victim-blaming is a wide-spread problem, and the strategy itself does not include efforts to understand or address victim-blaming. McClelland et al. (2020) highlight the power of public-facing surveys to perpetuate problematic ideas about already-stigmatized or marginalized individuals, and these two items do this in their reproduction of victim-blaming rhetoric. The use of myths in these items—with no effort to address them in the final strategy—therefore serves to perpetuate these myths, which has powerful consequences. In addition, these items were mired in contradiction. For instance, while the survey guidance stated that “There are no right or wrong answers, please give honest answers,” the items asked participants to indicate whether they thought statements were true or false, suggesting there was a “right” answer and a “wrong” answer. Therefore, rather than eliciting feedback on how to “tackle” violence, the items asked participants to take a position on these myths, and the use of the option “true” provided participants with an opportunity to buy into them. In addition, these items require participants to know about how violence plays out. Therefore, it is unclear as to whether these items measure their knowledge or their subscription to myths about violence.

### Contesting the Realities of Violence

In contrast with items that reproduced myths about violence, we also identified a converse scenario in which some fundamental realities of violence were called into question. Again, participants were asked to indicate whether they thought the following statements were true or false:

Violence against women and girls happens more often in a relationship between a man and a woman.

A person's race or religion can make them more likely to suffer from violence against women and girls.

Young people are more likely to suffer from violence against women and girls than older people.

Someone with a disability is more likely to suffer violence against women and girls than someone without a disability.

This again suggests that there was a “right” answer and a “wrong” answer. However, in this scenario, the items ask participants to take a position on these realities, and the use of the option “false” provides participants with the opportunity to reject these realities. However, the items are also doing another job here. In asking whether certain people are “more likely” to suffer violence, and whether violence occurs “more often” in certain relationship contexts, the items ask participants to give estimations of prevalence. Therefore, it is unclear as to whether the items are asking about the participants’ position on the realities of violence, or their knowledge about the *prevalence* of violence.

In addition, these are also blunt and de-contextualized statements, which essentialize violence along distinct and separate lines of identity. The wording of three of these items casts certain people as “more likely to suffer from” violence, and constructs identity characteristics within the person as risk factors. While this points to disparities in experiences of violence, it is not a person's individual characteristics or demographic per se that determines whether they experience violence. Rather, it is how power operates along the lines of identity and in a person's life or relationships. Indeed, as Hearn et al. (2016) note, “Violence is itself a form of social inequality, an unequal(izing) social structural division and relation of its own, a social distribution of who does what to whom. Violent practices link with social divisions and inequalities, class positions and other social intersections, as enactments of superordinate power, resistance to subordination and/or enactments of relative power in positions of subordination” (p. 553). And, rather than operating independently, power operates along these lines of identity simultaneously. Therefore, separating these identities reflects an overly simplistic approach to violence, which does not adequately capture the intersectional complexities of violence as it is brought to bear in the world. Locating violence in individual risk factors also functions to construct characteristics of the person, rather than constructed divisions and positionalities, as the reason for violence. Again, this risks reproducing victim-blaming rhetorics.

## Discussion

Our analysis echoes feminist researchers before us in arguing that quantitative research should produce socially valid and progressive knowledge and avoid the reproduction of patriarchal ways of knowing (Reinharz, 1992). One way to promote these goals is to examine the tools that are used in quantitative research (McClelland et al., 2020). In this article, we have presented an intersectional feminist analysis of a quantitative survey tool used by the UK government in order to generate evidence to inform their Tackling VAWG Strategy (HM Government, 2021), which provides a template for VAWG policy implementation. Based on the issues we have discussed regarding the conceptualization(s) of violence and the construction of survey items, we will now reflect on the issues we have raised to consider how a feminist approach might inform policy-based research. To do this, we will discuss some implications for quantitative research practices from a feminist perspective, and broader feminist methodological contributions that could address these implications.

### Implications for Quantitative Research Practices

Feminist scholars argue that surveys should generate data but also challenge existing power structures, question dominant narratives, and center the voices of marginalized groups (Reinharz, 1992). In relation to challenging existing power structures, Reinharz (1992) emphasizes the importance of writing survey questions that are sensitive to gender and power dynamics and inclusive of diverse experiences. This includes avoiding biased language, offering response options that reflect a range of identities and experiences, and acknowledging the social context in which respondents live. Our analysis shows that the government's survey did not challenge dominant power structures, and actually reinforced them. For instance, the survey items reproduced cisheteronormative assumptions about gendered violence and marked out racialized conceptualizations of sensationalized violence, such as honor-based violence. This served to privilege cisheteronormative identities and power relations and vilify certain perpetrators of violence over others. In addition, the survey promoted carceral conceptualizations of violence, which upheld this dominant structural response to violence without acknowledging its many shortcomings, including a failure to support survivors, the perpetuation of harm against survivors, and the disparate intersectional patterning of criminalization. To challenge existing power structures, Reinharz (1992) argues that quantitative analyses, including survey item analysis, can be conducted through a feminist lens that incorporates explorations of power dynamics, inequalities, and the intersections of gender, race, ethnicity, social class, and sexuality (Reinharz, 1992). We argue that this is only possible if the survey itself accommodates sensitivity to these issues. In our analysis, we found that the survey items prohibited space and scope for such analyses. On this basis, we argue that policy-related survey research should begin with a broad understanding of the power structures impacting the topic of interest and develop survey tools that can allow for adequate analyses of these structures and their disparate impacts. In the context of gender-based violence research and policy, a lack of attention to these power structures risks marginalizing those who are already marginalized by rendering them illegible, unknowable, and unprotected by policy (Cooper, 2015).

In relation to questioning dominant narratives, we found that rather than questioning dominant narratives, the survey instead reproduced a series of stereotypes and myths about violence. For instance, the conceptualizations of violence presented in the survey reproduced sensationalizing stereotypes about violence, depoliticized act-based depictions of violence, and rape myths that contribute to victim-blaming rhetoric. Quantitative research should avoid reproducing such problematic stereotypes and reductionist depictions of violence. To address these limitations, we argue that policy-based researchers should pay critical attention to contemporary stereotypes about violence in order to prevent the perpetuation of dominant narratives. In addition, researchers should avoid reproducing only dominant narratives about violence. For instance, instead of individualized act-based depictions of violence, a broader conceptualization could be developed to capture a spectrum of experiences, including psychological, emotional, financial, epistemic, and systemic violence.

In relation to centering the voices of marginalized groups, we found very little evidence of this in our analysis. Rather, as we have discussed, we found that the survey marginalized those groups even further. Feminist researchers have typically suggested solutions to this issue in the form of developing more purposive sampling strategies that promote diversity in a sample (e.g., Reinharz, 1992). However, if the survey itself does not integrate sensitivity to the experiences of marginalized groups, those groups will ultimately not be able to see themselves in the survey items. We recommend that quantitative policy-based researchers should explicitly consider the intersections of gender, race and ethnicity, sexuality, disability, and other relevant identities in relation to the topic of interest. In the context of gender-based violence policy research, survey instruments should use inclusive language and measures, avoiding binary categories where possible and phrasing questions in ways that do not reinforce stereotypes of violence or uphold patriarchal norms. Specifically, this should include special attention on the part of researchers to avoid constructing white, cisgender, and heteronormative experiences and relationship paradigms as normative.

### Feminist Methodological Contributions

Feminist researchers critique quantitative research on the grounds that it obscures nuanced participant experiences in favor of objectivity and standardization, at the expense of socially valid and progressive knowledge. Our analysis generated insights into a lack of objectivity and validity in the survey's construction, which has implications for the conceptualization and measurement of violence, as we have discussed. However, our analysis has also revealed a range of broader problems with the government's use of a quantitative survey, especially without the integration of critical and feminist considerations. We will now discuss some feminist methodological contributions that could address these problems.

Firstly, we have identified a broader problem with the marginalization of those who are already marginalized. In response, we argue that more complex research designs could usefully enrich research insights in this area. Specifically, we advocate for the development of qualitative research components. There are several qualitative methods that are valuable yet underutilized in comparison with surveys when exploring the topic of violence. These include feminist evaluation research, feminist ethnography, and social movement research (Hesse-Biber & Piatelli, 2012). In addition, surveys themselves can also integrate qualitative components, which can be highly effective in generating richer insights into the topic (Braun et al., 2021). Unlike quantitative surveys, which typically use closed-ended questions and are closely linked to the researcher's agenda, qualitative methods enable researchers to ask open-ended questions and follow-up probes, allowing participants to share their stories and perspectives in their own words (Beetham et al., 2021; Kelly & Westmorland, 2016). These methods provide a deeper understanding of the social, cultural, and structural factors that contribute to violence and impact victim-survivors. Through interviews and focus groups, researchers can explore the complex interplay of gender norms, power dynamics, socioeconomic factors, and other contextual influences on individuals’ experiences of violence (DeKeseredy & Dragiewicz, 2018).

Another valuable qualitative method is Feminist Participatory Action Research (FPAR); a collaborative research approach that aims to empower participants, center local knowledge, promote social change, and make silenced voices heard (Lykes & Hershberg, 2012). FPAR prioritizes the voices and experiences of marginalized individuals, including victim-survivors of violence, who are often excluded or silenced in mainstream research, such as survey research (Reid et al., 2006). By involving survivors as active participants in the research process, FPAR ensures that victim-survivors’ perspectives shape and drive the research agenda and center victim-survivors throughout. FPAR methods seek to empower participants by involving them in all stages of the research, from design to implementation to publication and circulation (Gatenby & Humphries, 2000). There is also emphasis on the importance of understanding violence within its broader social, cultural, and structural contexts, which can allow researchers to challenge structural power relations. Through participatory methods such as group discussions, storytelling, and community mapping, researchers and participants can explore the intersecting factors that contribute to violence, including gendered, racialized, and classed power dynamics (Reid & Frisby, 2008). Unlike surveys which often focus solely on data generation, FPAR aims to encourage social change by mobilizing communities and stakeholders, such as governments, to address the causes of VAWG rather than only recounting victim-survivor experiences (Reid & Frisby, 2008).

FPAR also offers principles that can be applied in quantitative research. We argue that these principles could help to address some of the problems we identified via our analysis. For example, regardless of method, a focus on collaboration with members of marginalized communities as expert consultants could address issues of marginalization. This participatory approach could support the research to address relevant issues and ensures the research is constructed to represent the voices of those most affected by gender-based violence. FPAR also invites critical attention to collaborative research practices, regardless of the methods utilized. For example, in gender-based violence research and policy-related settings, participatory approaches can address issues of over-research and research fatigue, which may lead to silence in the form of disengagement from research (Boesten & Henry, 2018; Neal et al., 2016; Sukarieh & Tannock, 2013). Finally, the focus on researcher positionality in FPAR could also prove useful for quantitative researchers to critically reflect on their own positionality and the ways their own identities, biases, and power may influence the research process. We suggest that researchers pay critical attention to their positionality and understandings of the research topic, especially when working with victims of gender-based violence who are already marginalized and/or minoritized. In addition, researchers should attend to biases in the research focus. For example, researchers should make efforts to address the persistent problem of ignoring the structural and social factors that contribute to violence in favor of individual and pathologizing factors.

## Concluding Comments

As we have argued, quantitative research methods are not necessarily at odds with feminist goals. However, it is important for feminist researchers to evaluate quantitative research in line with these goals. The UK government consultation discussed in this paper employed a survey design with largely quantitative items. We carried out this analysis in response to issues we encountered as feminist researchers engaging with this large-scale governmental policy-based research. While this analysis is specific to one particular research setting, we believe that the observations presented herein carry broader applicability in the domain of policy-related gender-based violence research. This is an important area for future research because the concerns of academic researchers have yet to be comprehensively integrated into research design and implementation in this policy-based domain.

We have drawn attention to several methodological concerns we identified in our review of this public consultation survey. These shortcomings relate to narrow, depoliticized, and stigmatizing conceptualizations of violence, and methodological issues pertaining to the validity and accuracy of survey items and the responses they elicit. Critical measurement analysis (McClelland et al., 2020) allowed us to critically examine the broader assumptions shaping conceptualizations of gender-based violence and the questions asked on this basis. We have also identified intersectional issues pertaining to who and what is made legible in these questions, and how this may inform policy in such a way that excludes those who are already marginalized, such as women of color and LGBTQIA+ individuals. As we have illustrated, engagement with feminist intersectional theory can allow researchers to meaningfully integrate intersectional analysis, which has been shown to be centrally important in gender-based violence research, especially in policy-related settings (e.g., Hearn et al., 2016). Combined, we argue that these concerns present important opportunities to develop feminist, intersectionally-informed research practices for gender-based violence research within policy-related settings. Future research could be enriched through meaningful engagement and critical consideration of the methods used to generate the evidence that informs policy.
